# The Impact of Dietary Habits on Sleep Deprivation and Glucose Control in School-Aged Children with Type 1 Diabetes: A Cross-Sectional Study

**DOI:** 10.3390/children11070779

**Published:** 2024-06-27

**Authors:** Merve Askin Ceran, Muteber Gizem Keser, Murat Bektas, Nurhan Unusan, Beray Selver Eklioglu

**Affiliations:** 1 Department of Pediatric Nursing, Health Science Institute, Dokuz Eylul University, İzmir 35340, Türkiye; ms.cerancer3642@gmail.com; 2 Vocational School of Health Services, KTO Karatay University, Konya 42020, Türkiye; 3 Department of Nutrition and Dietetics, Faculty of Health Sciences, KTO Karatay University, Konya 42020, Türkiye; nurhanunusan@gmail.com; 4 Department of Pediatric Nursing, Faculty of Nursing, Dokuz Eylul University, İzmir 35340, Türkiye; mbekta@gmail.com; 5 Division of Pediatric Endocrinology, Faculty of Medicine, Necmettin Erbakan University, Konya 42090, Türkiye; berayselver@hotmail.com

**Keywords:** children, glucose control, nutrition, sleep, type 1 diabetes

## Abstract

Diet plays a crucial role in managing type 1 diabetes (T1DM). **Background/Objectives**:This study aimed to determine the impact of nutritional habits on sleep deprivation and glucose control in school-aged children with T1DM. **Methods:** In this cross-sectional study, nutritional habits and sleep deprivation were assessed in 100 school-aged children with T1DM, aged 7–13 years. The Dietary Habits Index and the Sleep Deprivation Scale for Children and Adolescents were used to evaluate nutritional habits and the level of sleep deprivation. Patients’ sociodemographic and nutritional variables were collected through researcher-composed questionnaires. HbA1c levels over the past 6 months were obtained from the patient data system. **Results:** The study found a moderately strong positive correlation between the Dietary Habits Index score and HbA1c (*p* < 0.001), with 28% of the variation in HbA1c explained by changes in the Dietary Habits Index score. However, no correlation was found between the Dietary Habits Index score and the level of sleep deprivation. **Conclusions:** The nutritional habits of school-aged children with T1DM may affect glucose control and sleep deprivation. Therefore, it is important to educate children with T1DM on making healthy food choices to manage their condition effectively.

## 1. Introduction

Type 1 diabetes (T1DM) is an autoimmune disorder caused by the destruction of B cells and deficiency of insulin. In 2022, it was reported that out of 143,396 diabetics, 29,000 were aged ˂20 years [1]. According to the International Diabetes Federation Diabetes Atlas, there were 8.75 million people with type 1 diabetes globally in 2022. The peak incidence occurs at ages 5–9 years in girls and 10–14 years in boys [1]. Numerous environmental factors are significant triggers of T1DM, with diet and microbiota effects on inflammation being two primary contributors.

Diet is one of the major cornerstones in the management of diabetes. The primary goal of a healthy and balanced diet is to control weight, maintain normal blood glucose levels, prevent complications due to high or low blood glucose levels, and ensure proper growth. An appropriate eating pattern includes main meals and snacks with regular mealtimes [2]. Dietary recommendations for children with diabetes are the same as those for healthy children. According to the International Society for Childhood and Adolescent Diabetes guidelines, the daily energy intake should be 45 to 50 per cent from carbohydrates, 30 to 35 per cent from fat (saturated fat < 10 per cent), and 15 to 20 per cent from protein [3]. However, some school-aged children with diabetes do not meet the nutritional requirements. A healthy, balanced diet includes consuming low glycemic index foods, reducing dietary cholesterol and saturated fat, and increasing the intake of fruits, vegetables, and whole grains [4]. In addition to macronutrients, micronutrients are crucial for a balanced diet, particularly during childhood, which is a critical phase for growth. Poor dietary choices can lead to health issues such as anemia, growth retardation, and anorexia due to vitamin and mineral deficiencies. A Mediterranean-style diet may be beneficial for managing diabetes and any other inflammatory diseases [5,6,7]. Microbial diversity and gut microbiota are other key factors in the development of diabetes. A healthy gut barrier prevents the entry of harmful substances into the bloodstream, reducing the risk of inflammation and immune system activation [8]. The gut microbiota interacts with diet and influences health outcomes [9]. Intestinal permeability and barrier dysfunction can trigger the onset and progression of T1DM, affecting the immune system and activating inflammation. Diet influences gut microbiota diversity, particularly in butyrate-producing communities [10].

Another factor that affects the health of diabetic children is sleep. Sleep is essential for improving attention, behavior, memory, emotional regulation, and overall quality of life. For school-aged children, the recommended average sleep duration is 9–12 h per day [11]. Poor sleep in childhood can predict future obesity, depression, and cardiovascular diseases [12,13]. Insufficient sleep or poor sleep quality can also worsen glucose control in diabetic patients, with higher HbA1c levels observed in those with inadequate sleep [14,15,16,17]. A review of the literature revealed no studies investigating the effect of dietary habits on sleep and HbA1c levels. Therefore, the aim of this cross-sectional study was to evaluate the impact of dietary habits on sleep deprivation and glycemic control in school-aged children with T1DM.

## 2. Materials and Methods

### 2.1. Design and Setting

This descriptive, correlational, and cross-sectional study was conducted between September 2023 and February 2024. Children aged 7–13 years who were diagnosed with type 1 diabetes and followed by Necmettin Erbakan University, Meram Medical Faculty, Department of Child Metabolism, were enrolled in the study.

The required sample size was calculated to be 68 participants, based on a regression analysis using the G-power program, with a significance level of 0.05, 80% power, and a medium effect size for regression. To ensure robust results, 119 children who were being followed up in the clinic were initially included. However, 6 children were excluded due to missing records, and 13 were excluded because they did not fall within the specified age range. Consequently, the final analysis evaluated the results of 100 patients. All children included in the study had been previously diagnosed with type 1 diabetes.

### 2.2. Variables and Measurements

Sociodemographic variables, presence of any other disease, physical activity level, diabetes duration, frequency of blood glucose control, and related data were collected via a researcher-administered questionnaire (Supplementary File). Data were collected through face-to-face interviews. The weight and height of children were measured at the hospital by researchers. Body mass index (BMI) was calculated according to WHO formula: weight/(height × height). The children’s mean HbA1c level over the past 6 months was accessed from the patient data system at the hospital by one of the researchers, who is a pediatric endocrinologist. Additionally, the Sleep Deprivation Scale for Children and Adolescents and the Dietary Habits Index were administered to school-aged children with type 1 diabetes.

#### 2.2.1. Sleep Deprivation Scale for Children and Adolescents

Kandemir et al. (2021) developed a scale that aimed to determine the sleeplessness level of children and adolescents [18]. The scale consists of 15 items on a Likert scale (ranging from “agree” to “disagree”). Scores on this scale range from 15 to 60 points. Higher scores indicate worse sleep deprivation. The analysis for sampling adequacy yielded a Kaiser–Meyer–Olkin (KMO) value of 0.94, and the Bartlett test result was χ^2^ = 1833.03 (*p* < 0.001). Exploratory factor analysis (EFA) indicated that the scale has a single factor structure, explaining 54.48% of the variance, with an internal consistency reliability coefficient of 0.94. Confirmatory factor analysis (CFA) was then conducted to verify the one-dimensional structure. The CFA results showed that Chi-Square/degrees of freedom was 254.94/65, 3.92; the RMSEA value was 0.07, and the RMR value was 0.027. The fit indices for the tested model were CFI = 0.94, GF3 = 0.91, AGFI = 0.91, IFI = 0.96, NFI = 0.94, and TLI = 0.97. Cronbach’s alpha internal consistency value was found to be 0.94.

#### 2.2.2. Dietary Habits Index

Dietary habits were assessed using the 6-item Dietary Habits Index, which was developed by Demirezen E. (1999) and revised in a 2005 study [19,20]. The risk level of dietary habits was evaluated based on the total score obtained from the Dietary Habits Index. Scores on this index range from 0 to 24 points. According to the assessment criteria, a score of 0 indicates no nutritional risk, a score of 1 to 6 indicates a low nutritional risk, a score of 7 to 12 indicates a moderate nutritional risk, a score of 13 to 18 indicates a high nutritional risk, and a score of 19 to 24 indicates a very high nutritional risk. The Cronbach’s alpha internal consistency value for this index was found to be 0.28.

### 2.3. Data Analysis

Data analysis was performed using the SPSS 24.0 program (SPSS, Chicago, IL, USA). Descriptive data were presented as frequency, arithmetic mean, minimum, maximum, standard deviation, and percentage. The effect of dietary habits on sleep deprivation and glucose control was evaluated using multiple regression analysis. Multicollinearity was examined using the variance inflation factor (VIF) and tolerance value. It was determined that VIF values were less than 10 and tolerance values were greater than 0.2, indicating no multicollinearity. The relationship between dietary habits, sleep deprivation, and glucose control was assessed using Pearson’s correlation analysis. The level of statistical significance was set at *p* < 0.05.

### 2.4. Ethical Considerations

The study was conducted in accordance with the Declaration of Helsinki. Ethical approval was obtained from the Medicine Faculty Ethics Board of KTO Karatay University (Number: 2023/037, Date: 21 September 2023). Additionally, permission was obtained from the university where the study was conducted. Informed consent was obtained orally from all the children and in writing from their parents.

## 3. Results

In this study, 53% of the children were female, and the mean age was 10.14 ± 1.79 years. The mean weight was 40.01 ± 11.73 kg for males and 36.78 ± 10.14 kg for females. The mean height for the children was 140.79 ± 14.27. Among the children, 26% had other health problems, with coeliac disease being the most prominent, diagnosed in 18 children. Regarding the children’s diabetes characteristics, the mean duration of diabetes was 2.81 ± 2.01 years, and the mean age of diagnosis was 7.05 ± 2.21 years (Table 1).

The mean score of the Dietary Habits Index among school-aged children with type 1 diabetes was 11.59 ± 3.36, indicating a moderate risk. It was found that 46 children fell into the medium-risk group with scores between 7 and 12 (Table 2).

The mean score on the Sleep Deprivation Scale for Children and Adolescents was 32.14 ± 11.46 (Table 2).

There was a moderately strong positive correlation between the Dietary Habits Index score and HbA1c level. Additionally, a positive but weak correlation was found between the Dietary Habits Index score and the Sleep Deprivation Scale for Children and Adolescents score. No significant relationship was found between sleep disturbance and HbA1c (Table 3).

The regression coefficient for the variable in the model was found to be 4.876, with a standardized regression coefficient of 0.528. This indicates that increasing the Dietary Habits Index score can reduce HbA1c levels by an average of 0.289. Furthermore, 28% of the variation in HbA1c was explained by changes in the Dietary Habits Index score (Table 4).

The regression coefficient for the variable in the model was 19.907, with a standardized regression coefficient of 0.307. This indicates that an increase in the Dietary Habits Index score can increase the Sleep Deprivation Scale score by an average of 1.057 (Table 5).

## 4. Discussion

More than 1.1 million children and adolescents worldwide are being followed up with a diagnosis of T1DM. More than 86,000 children under 15 years of age develop T1DM annually. A study conducted in 2013 found that the incidence in our country was 10.8 per 100,000 per year [21]. From the moment of diagnosis, children, adolescents, and their parents are responsible for intensive and complex diabetes management, which requires constant attention and effort [22]. Diabetes management involves regular blood glucose monitoring, multiple insulin injections, maintaining a healthy diet and activity level, adjusting insulin doses to suit life patterns, and regular hospital visits [22,23]. The goal of managing children with type 1 diabetes is to ensure continued growth and development while achieving optimal glycemic control. This approach protects the child from acute and chronic complications. Dietary habits play a crucial role in ensuring proper nutrition. Given this context, a cross-sectional study was designed to examine the impact of dietary habits on sleep deprivation and glucose control among school-aged children with T1DM.

In this study, the mean score of the Dietary Habits Index indicated a moderate nutritional risk. One of the main findings is that the dietary habits of school-aged children with type 1 diabetes can significantly impact blood glucose control. A moderate and significant correlation was found between the Dietary Habits Index and HbA1c levels, suggesting that an increase in the Dietary Habits Index score can reduce HbA1c levels by an average of 0.289. This study found a moderately strong positive correlation between the Dietary Habits Index score and HbA1c levels.

A study by Seckold et al. included 22 children aged 4.9 ± 1.3 years with an HbA1c of 6.4% ± 0.9% [24]. The study concluded that the quality of diet is a concern in young children with T1DM, characterized by excessive saturated fat and inadequate vegetable intake. Similarly, Tayyem et al. identified dietary patterns associated with glucose control in 107 children and adolescents with T1DM. The study revealed that only 25.7% of the participants had good glycemic control. Overall, three dietary patterns were identified: “High-Vegetables”, “Unhealthy”, and “High-Fruits”. The “High-Vegetables” dietary pattern demonstrated a protective relationship in controlling HbA1c levels, particularly in the second and third tertiles [25].

The traditional Mediterranean diet is characterized by a high consumption of vegetables, fruits and nuts, legumes, and unprocessed cereals, and a low consumption of meat, meat products, and dairy products. A study conducted by Dominguez-Riscart et al. (2022) reported improved HbA1c levels in 97 individuals with type 1 diabetes who adhered optimally to the Mediterranean diet [26]. Similarly, a study aimed at examining changes in diet composition over 10 years in children and adolescents with type 1 diabetes involved 229 participants aged 6 to 16 years. The study found that the diet composition of these children and adolescents changed, leading to improved neurometabolic control [27].

Bodur et al. (2021) conducted a cross-sectional study of adolescents (10–19 years old) with type 1 diabetes, showing a weak and negative relationship between the diet quality scores of the male participants and their waist circumference and HbA1c levels (*p* < 0.05) [28]. Nansel et al. (2016) investigated the association between dietary intake and several indicators of blood glucose control in 136 adolescents with type 1 diabetes participating in an educational nutrition intervention. They found that both the overall quality of the diet and the distribution of macronutrients were associated with more optimal glycemic control [29]. Conversely, Zhou et al. (2021) found that daily food intake did not affect glucose control in adults with type 1 diabetes, but C-peptide levels influenced glycemic variability independently of mean blood glucose [30].

Most studies have focused on individuals with type 2 diabetes, with comparatively less research examining sleep in those with type 1 diabetes [31]. The interaction between sleep parameters and type 1 diabetes is crucial, as blood glucose control plays a significant role [32]. In a systematic review of sleep and type 1 diabetes, adults with type 1 Diabetes reported poorer sleep quality but not shorter sleep duration compared with adults without diabetes [17]. Another study found a significant relationship between increased HbA1c levels and the prevalence of sleep disorders, suggesting that sleep disorders can increase HbA1c levels and may be a risk factor for increased levels of HbA1c [33]. Jaser et al. conducted a study with 2- to 12-year-old participants with type 1 diabetes and found that 67% of children met the criteria for poor sleep quality. Sleep quality was associated with glycemic control but not with average glucose monitoring [34].

This study aimed to determine the effects of dietary habits on HbA1c levels and sleep deprivation in school-aged children with type 1 diabetes. It is known that sleep deprivation increases as the score obtained from the scale increases. A moderate and significant correlation was found between the Dietary Habits Index and Sleep Deprivation Scale for Children and Adolescents (*p* < 0.05). The study revealed that an increase in the Dietary Habits Index score could lead to an average increase of 1.057 in the sleep deprivation score. The change in the Dietary Habits Index score accounted for 0.9% of the variation in the sleep deprivation score.

Conversely, a study by Brandt et al. (2020) involving 20 people with type 1 diabetes found no association between sleep quality and the time spent in the target glucose range or above or below the target glucose range [35]. Similarly, Corrado et al. (2023) conducted a cross-sectional study with 120 adults with type 1 diabetes and found no differences in postprandial blood glucose levels between participants with poor and good sleep quality [36].

The study has certain limitations due to the nature of the sample and the methodology employed. Firstly, the study was designed as cross-sectional. Additionally, dietary habits were determined using a scale rather than food frequency records of actual food consumption. Despite these limitations, the significance of this study lies in assessing the effects of dietary habits on both glycemic control and sleep deprivation among school-aged children with type 1 diabetes.

## 5. Conclusions

The data indicate that dietary habits not only affect HA1c levels but also the degree of sleep deprivation in children with type 1 diabetes. It is important to remember that the dietary habits of school-age children are influenced by many environmental factors. Therefore, healthcare professionals, particularly dietitians, should regularly assess the dietary habits of children with type 1 diabetes and provide guidance on recommended dietary practices. In addition to nutritional advice from health professionals, web-based education or mobile applications may be recommended to provide both children and parents with up-to-date information on nutrition in type 1 diabetes. Further prospective, randomized, large-sample research is needed to confirm the potential therapeutic implications and longer-term outcomes. Additionally, the effects of dietary habits on sleep deprivation and glucose control in children of different age groups with type 1 diabetes should be examined in further studies.

## Figures and Tables

**Table 1:** Descriptive characteristics of the children (n = 100).

Characteristics	n (%)/Mean ± SD	Min–Max
**Gender**		
Female	47 (47)	
Male	53 (53)	
Age (year)	10.14 ± 1.79	7.00–13.00
Height (cm)	140.79 ± 14.27	105–170
BMI (kg/m^2^)	19.02 ± 3.96	12.98–44.62
Education level		
No education	1 (1)	
Primary school	34 (34)	
Secondary school	65 (65)	
Physical activity level		
None	23 (23)	
Lower than 30 min	32 (32)	
More than 30 min	45 (45)	
HbA_1c_ (%)	8.28 ± 1.82	5.90–13.00
Duration of Diabetes (year)	2.81 ± 2.01	0–10
Age at diabetes diagnosis	7.05 ± 2.21	2–11
Presence of diabetes in the family		
Yes	60 (60)	
No	40 (40)	
The degree of proximity		
First degree	28 (28)	
Second degree	30 (30)	
Third degree	1 (1)	
Fourth degree	1 (1)	
Presence of other diseases		
Yes	26 (26)	
No	74 (74)	
Names of other diseases		
Hypertension	1 (3.8)	
Celiac disease	18 (69.2)	
Other	7 (27)	
Frequency of blood glucose monitoring		
Once a day	6 (6)	
Twice a day	61 (61)	
Three times a day	7 (7)	
Every hour	3 (3)	
Every 30 min	23 (23)	
Meal time a day		
Total number of meals	5	3–6
Number of main meals	3	2–4
Number of snacks	2	0–4

HbA1c, glycated hemoglobin A1c; M, mean; SD, standard deviation.

**Table 2:** Mean scores of Dietary Habits Index and Sleep Deprivation Scale for Children and Adolescents.

Scales	M ± SD	Min–Max
Dietary Habits Index Score	11.59 ± 3.36 *	3–18
Sleep Deprivation Scale for Children and Adolescents Score	32.14 ± 11.46	15–60

* This score is valid in 97 participants; M, mean; SD, standard deviation.

**Table 3:** The relationship between scale scores and Hba_1c_ levels in school-aged children with type 1 diabetes.

Variables		Dietary Habits Index Score	Sleep Deprivation Scale for Children and Adolescents Score	HbA_1c_
Dietary Habits Index Score	r	1		
	*p*			
Sleep Deprivation Scale for Children and Adolescents Score	r	^a^ 0.307	1	
	*p*	* 0.002		
HbA_1c_	r	^a^ 0.528	^a^ 0.181	1
	*p*	** <0.001	0.075	

^a^ Pearson coefficient; * *p* ˂ 0.05; ** *p* ˂ 0.001.

**Table 4:** The prediction of Dietary Habits Index score on HbA1c Level in school-aged children with type 1 diabetes.

Variables	Beta	Standard Error	β *	t **	*p*	95% CI	
						**Lower**	**Upper**
Constant	4.876	0.585		8.340	0.000	3.715	6.036
Dietary Habits	0.289	0.048	0.528	5.996	0.000	0.194	0.385
R ***	0.528						
R^2^ ****	0.279						
F *****	35.952						
*p*	<0.001						
DW	1.776						

β *, standardized beta; t **, *t*-test value; R ***, correlation coefficient; R^2^ ****, R square; F *****, ANOVA value.

**Table 5:** The prediction of Dietary Habits Index score on the Sleep Deprivation Scale for Children and Adolescents score in school-aged children with type 1 diabetes.

Variables	Beta	Standard Error	β *	t **	*p*	95% CI	
						**Lower**	**Upper**
Constant	19.907	4.052		4.913	0.000	11.863	27.952
Dietary Habits	1.057	0.336	0.307	3.146	0.002	0.390	1.724
R ***	0.307						
R^2^ ****	0.094						
F *****	9.895						
*p*	>0.001						
DW	2.270						

β *, standardized beta; t **, *t*-test value; R ***, correlation coefficient; R^2^ ****, R square; F *****, ANOVA value.

## Data Availability

The data presented in this study are available on request from the corresponding author due to privacy restriction.

## Supplementary Materials

The following supporting information can be downloaded at: https://www.mdpi.com/article/10.3390/children11070779/s1, The Impact of Dietary Habits on Sleep Deprivation and Glucose Control in School-Aged Children with Type 1 Diabetes: A Cross-Sectional Study Questionnaire.
