# Supplementary material for: The Impact of Dietary Habits on Sleep Deprivation and Glucose Control in School-Aged Children with Type 1 Diabetes: A Cross-Sectional Study
Source: Children (Basel). 2024 Jun 27;11(7):779. doi: 10.3390/children11070779 (PMC11276351; doi:10.3390/children11070779)
Supplement: Supplementary file 1 [file children-11-00779-s001.zip › children-3008040-supplementary.pdf]

The Effects of Nutrition Habits on Sleep Deprivation and Glucose Control in School Age Children with Type 1 Diabetics: A Cross-Sectional Study Questionnaire

This study is being conducted to evaluate the effect of nutritional habits of children with type 1 diabetes on sleep deprivation and glucose control. The information obtained from the survey will not be used for any purpose other than scientific publication. Participation in the survey is voluntary and no personal information of the survey participant is requested. Thank you for your participation.

Anket no:

A) Sociodemographic Information

1. Birth Date:
2. Gender: Male ( ) Female ( )
3. Weight ………………..kg Height:………… cm
4. Education level: Not going to school ( ) Primary education ( ) Secondary education ( )
5. Physical activity level during the day: Never ( ) Lower than 30 min ( ) Up to 30 min ( )

B) Questions About Diabetes

6. Diabetes diagnosis age ……………
7. Diabetes age ………… (year)
8. Any other diseases other than diabetes (If your answer is no, go to question 10.)

   Yes ( ) No ( )

9. What disease(s) do you have? (You can select multiple options)

   Hypertension ( ) Kidney Disease ( ) Other ( ) ….

10. Do any family members have diabetes?

    Yes ( )…………..degree No ( )

11. How often do you measure your blood sugar?……………………………………..

C) Questions About Nutrition

12. Total number of meals per day :
13. Number of main meals per day :
14. Number of snacks per day:
15. If you are taking meals, please state the reason.

Because I couldn't wake up in the morning ( ) For weight loss purposes ( ) Because of time constraints ( ) Because I couldn't think of it ( ) Because there was no one who prepared it ( ) Because I had no appetite/didn't feel like it ( ) Others ( )……………..
